# The Gheel Question

**Published:** 1861-07

**Authors:** J. Munday

**Affiliations:** of Moravia.


### Art. VI.—THE GHEEL QUESTION.

By J. Munday, M.D., of Moravia.

*"Et tamen movetur."*

The *Psychological Journal* has at various times admitted articles on Gheel, but since last year it has been so *tolerant* as to open its pages to the "pros" and "cons" of this important question of mental science.* We have said "tolerant," not without a meaning, as we have unfortunately experienced that some journals *systematically refuse* every article in defence of Gheel, yet argue in every number against the colony. We wish it to be understood that this charge is particularly directed against the *Allgemeine Zeitschrift für Psychiatrie*, published by Hirschwald, of Berlin, and refers especially to recent articles on Gheel. The author of these articles is the Geheimrath Dr. Flemming, of Schwerin, formerly director of the celebrated asylum on the Sachsenlerge, near Schwerin, a gentleman who has not only distinguished himself by his practical abilities, but has made a name in our science by his splendid writings. If, notwithstanding the position of this high priest in the art of Phrenopathy, we undertake to write against his views, we who have hardly entered the portals of this temple of science, it will necessarily be our endeavour to deal only with the question at issue, and not with the person. We do not feel unqualified for this undertaking, as we have devoted many years to this especial subject, and have been on several occasions at Gheel, and the last time spent several months there occupied in theoretical and practical studies. We do not intend to enter into the criticisms which Dr. Flemming has published on the articles of Professor Parigot, of Brussels.† To some of these we have only lately written a reply.‡ We shall also not discuss the communication which Dr. Willers Jessen addressed to this journal, and we shall treat with silent disdain the "Dymphna" history, which has really become intolerable.§ We have already expressed our views in reference to the opinions of our esteemed

* Refer to the January part of 1857, and the July number, 1860, of this Journal, pp. 277 to 291; and again, October part, 1860, pp. 600, 602, and 612.

† Refer to the *Allgemeine Zeitschrift für Psychiatrie*, 1860, vol. xvii., part iii., p. 366; part iv., p. 751; and part v., p. 805.

‡ Refer to the *Correspondenz Blatt der deutschen Gesellschaft für Psychiatrie* Neu-Wied, 1860, No. xix. and xx., p. 300.

§ Refer to the October part, 1860, of this Journal, p. 612.

English confederates, Drs. Brown and Bucknill, in other places.* If we therefore again throw down the gauntlet to our friends beyond the Rhine, we do it, not for the purpose of disputing mere phrases which appear to us trivial,† but to deal with subjects, the importance of which is still too much undervalued, because as yet misunderstood. We only hope that our antagonists will not desist from controversy with us, but will readily enter into a question which can only be decided by a thorough investigation. These few words may be looked upon as an introduction to our article, whose object is chiefly to comment on those questions which Dr. Flemming forwarded to the general meeting of German physicians which assembled at Eisenach on the 12th and 13th of September, 1860, having been ourselves prevented by illness from appearing there personally. We shall presently quote the wording of the questions laid before the meeting, as also the replies which were written down by the secretary, Dr. Heinrich Laehr, and published in a separate report which was circulated in the *Allgemeine Deutsche Zeitschrift für Psychiatrie,* 1860, vol. xvii. part 617. As we were not present at this meeting, we regret that the discussion of these questions has been entirely omitted, and only the summary replies communicated. As far as we can understand, it would appear that great objections were made to non-professional gentlemen entering into the discussion. This doubtless refers to M. Jules Duval, of Paris, as no other person out of the medical profession has, to our knowledge, ever made a serious study of this question.‡ We necessarily exclude articles in newspapers, and other popular journals, but M. J. Duval is a non-professional gentleman of an entirely exceptional cast. Forgetting for a moment that this celebrated Frenchman is one of the most esteemed writers in the *Journal des Débats* and the *Revue des Deux Mondes,* he possesses the great advantage of having personally examined the subject, for he has visited Gheel, a thing his antagonists have hitherto neglected to do. M. Duval is, besides, a philanthropist, endowed with great experience, who has practically laboured in this field of science, a man of extended philosophical education, combined with a warm heart, and an untiring industry in all that

---

* Refer to the *Deutsche Klinik,* 1858, Nos. xix. and xx.; further, to the *Allgemeine Zeitschrift für Psychiatrie,* 1859, vol. xvi., p. 442; compare with it our two articles in the *Journal de Médecine à Bruxelles,* May, 1860, p. 451, and August, p. 220; further, the *Asylum Journal,* April, 1858, p. 202, and January, 1859; also *Revue des Deux Mondes,* November 1, 1857; and, lastly, *Allgemeine Zeitschrift für Psychiatrie,* 1858, vol. xv., p. 412.

† This refers to a criticism of Dr. Flemming on the translation of the word "Flitterstaat," wrongly rendered by "frivolité," better by "faux brillant;" compare *La Presse Médicale Belge,* 1860, No. xxxv., August 19, p. 277, and note 2 of this article, the last quotation, as well as note 3 of this article.

‡ Refer to Jules Duval's *Gheel une Colonie d'Aliénés vivants en Famille et en Liberté.* Paris, 1860. Guillaume et Cie.

is noble and elevated—these qualifications indubitably give him a right to be heard. So much on the subject of this estimable non-professional gentleman. We must add, that we are of the number of those who are ready to enter the lists with the *dilettanti* in our science, and are always anxious to avoid appeals to public clamour, if our antagonists be only content to fight with more equal weapons. The following are the questions Dr. Flemming forwarded to the Eisenach meeting:—

1. Is it still a pressing question whether the asylums of the various states and provinces of Germany, with their present population, are judiciously managed and sufficiently cheap to keep the incurable, or detain those lunatics who may require it?

2. Are we to consider the discussion closed on the much recommended imitation, as a relief to the asylums, of the Belgian colony for lunatics at Gheel, which is repeatedly urged by Belgium, and are we to consider the question rejected?

3. Are we to wait for final information on the last assertion of Parigot, that, according to the number of recoveries in the colony for lunatics, the most favourable conclusions may be drawn as to their cure?

4. If the questions 1 and 2 are affirmed, how can the requirements of the public, of sanitary police, and humanity be best satisfied, in reference to the keeping of the patients, and what kind of financial relief can be given to the states?

Either—

*a*. By the proposition of a plan for more simply constituted and less costly asylums; or

*b*. By the creation of a colony for lunatics relatively united beyond the frontiers of their states.

What principles should be adopted for their guidance?

The meeting thus replied to these questions:—The first question regarding the best and cheapest method of keeping the incurable, and detaining the other lunatic patients, is still a very important and pressing one: the imitation of the colony for lunatics at Gheel, recommended as a relief, is to be rejected: the experience of the last ten years has proved that it is possible to reduce the building expenses of lunatic establishments by adopting a simpler style of architecture: it is desirable in building an asylum to profit by the advice of a scientific Committee of Physicians: that with reference to the question of colonies in conjunction with these establishments, it shall not yet be rejected, but be kept open, to be more fully discussed with new propositions to be brought forward at the next meeting.

We will now endeavour to comment on these questions analytically.

1. What alienist physician could give a negative to this question? There is a general complaint all over Germany, which can be daily heard in the asylums, of the increasing lunatic population. Almost all German asylums are trying to increase their space by additional buildings. In almost all German States there are either new buildings for these purposes commenced or they are projected. In Austria alone, 5; in Prussia, 2; in Hanover, 1. It is not surprising, therefore, that this question should have been affirmed, but it is surprising that it should have been necessary to put it at all in face of these facts. It seems that the question has been raised chiefly as a basis to the following:—that this is a fact, not only in Germany, but all over the world. In Holland, the country we acknowledge as the model of our department, we find the great Schröder van der Kolk at present occupied with the plan of a central asylum for all incurable cases, as the lunatic population in all asylums of Holland has "increased" enormously. Belgium possesses fifty asylums, and only one Gheel; but all those in whose hands the management of this subject rests look to the latter, and are anxious to find a remedy by establishing a second Gheel in Ardennes. In France we find every year an increase of both private and imperial asylums for lunatics. Russia is much occupied at present with a complete reorganization of its asylums; such is also the case in Denmark, Sweden, and Norway. Spain sanctioned a competition of the whole world to project a new model establishment near Madrid, and sent commissioners all over Europe, in order to gain information on the subject. Switzerland is now building new asylums. Italy and Portugal complain of their insufficient and antiquated arrangements, and are anxiously wishing to reform them. England, which formerly concentrated this branch too much, now seeks to separate it, and the lunatic physicians of Scotland personally acquainted themselves at Gheel with the principles necessary for the future organization of their own lunatic asylums.* In the states out of Europe, including Turkey, which to this day remains indifferent as to the welfare of lunatics, we find, both in North and South America, only private asylums, large and small, and merely erected to gain money by their scientific arrangements. Grand new palaces (*hôtels garnies prisons*) are daily established, which are called asylums. There are, however, some respectable institutions, and creditable exceptions.† Holland is trying, through the endeavours of the

* The following Scottish physicians have visited Gheel:—Dr. Coxe (twice), Dr. Mitchell, and Dr. Sibbald, all three from Edinburgh. Dr. Browne, one of the Commissioners in Lunacy, of that country, also published his views on Gheel in the *Daily Scotsman*, September 5th and 11th, October 11th, 1857, and *Asylum Journal*, April, 1858, p. 202.

† Refer to the *Report of the Eastern Lunatic Asylum, in the City of Williamsburg, Virginia*, 1858, pp. 56, 57. Richmond, printed by Bischeaud et Dunavant, 1857.

gentleman already mentioned, to introduce into its colonies the new system of improvements. We have thus stated in a few words founded on personal observation and experience, as well as based on historical information, that there can be no doubt respecting the affirmation of the first question. We are well acquainted with the trying and difficult position of the Directors of Asylums in Germany and elsewhere, regarding their administration and the experience attached to it in reference to the government, and the financial committees, and administrative government building surveyors. We can, however, only glance at this part of the question without entering on a special discussion of it, as it does not strictly belong to the subject; but we find in all these points a pressing reason for affirming the proposed question. On a more minute examination we are driven to the second portion of the same question—*i.e.*, "how to keep the incurable, and detain those lunatics who require it." This phrase is for us a question involving a great principle. We ask, what curable or incurable lunatic requires detention? We reply, detention or seclusion can only have this double aim:—1st. To protect society at large and the patient himself from danger in general, and prevent self-destruction of every kind. 2nd. A therapeutic aim, by separation, discipline, &c. Where this aim is not obtained, or where it is not positively demanded, all detention of a lunatic is an *irrational barbarous abuse.* This is, alas! still followed from prejudice, power of habit, idle fear, routine, and sometimes also from baser motives. The consequences of which, openly pronounced, are these, that in the year 1861 no less than 125,000 lunatics, nearly half the entire lunatic population of Europe, are unjustly condemned to detention. It would lead us too far away to illustrate this fundamental question in detail here, we will endeavour to do this in a larger work, which, although we are constantly engaged in its composition, cannot be soon finished on account of the wide area it embraces.* We will, therefore, now leave the first question as settled, and enter on the second.

2. The meeting at Eisenach decided on rejecting this question. But we must be allowed to ask, is it justifiable, or even permissible, thus finally to reject an objective question in science of this kind, and close the discussion? Further, have we not a right to look upon such a rejection as an act which can carry with it no authority whatever? What would become of science if we were to dispose of important questions in this summary manner, and not admit dissent and discussion?

* We have been occupied for a lengthened period with the composition of a larger Phrenopeutic work, with the motto, "Incedo per ignes."

Truly we should be driven to the *et tamen movetur* of the martyr Galileo. The two questions noted have only been twice publicly and verbally discussed in societies by alienist physicians, and these discussions are of very recent date. The Eisenach meeting discussed the question on the 12th September, 1860; and the Medico-Psychological Society of Paris discussed it during its meeting on the 26th June, 1860, on the motion of M. Brierre de Boismont, and afterwards recurred to it in the following meeting on the 30th July, by desire of M. Moreau de Tours. At present we intentionally forego a critical examination of this discussion, and express only our gladness at the final result of the Paris meeting of the 30th of July, in which it was resolved, on the motion of the President, M. Trélat, seconded by M. Archambault, that Messrs. Michea, Moreau (de Tours), Mesnet, J. Falret, and Ferrus, should be requested to do that which was most palpably best, and what had been done by Esquirol and Voisin on the 21st August, 1821, namely, to proceed to Gheel, and to report their personal observations to the society. Let us hope that these gentlemen will not make so short a stay at Gheel as Esquirol and his distinguished disciple did; for forty hours' stay at Gheel would not now be a sufficient time in which to furnish a critical report on the institution; we who have been several months there may be permitted to have an opinion on the matter. Hitherto this colony for lunatics has only been criticized by gentlemen who have either not been there at all, or who have scarcely devoted twelve hours to its examination, out of which they have rested and refreshed themselves, and given but two hours to the "Patronalem Asyle" itself; and be it remembered that the colony contains nine French miles, is situated in fourteen districts, and has a town with 11,206 inhabitants, of whom 1000 are lunatics. We think it interesting and important to give the names of every visitor, both medical and philanthropical, who has visited Gheel during the last five years up to December, 1859; and this is after the period in which the reorganization of Gheel began.

From *Belgium*, Guislain, Ducpetiaux, Parigot, Theis, Perkins, Bull, Onez, Sauveur, Koepel.

*Holland.*—Schröder van der Kolk, Veith.

*Russia.*—Leifert, Konowishe, Babienski, Lorenz, Arneth.

*Sweden.*—Ohiström; and Dall, from *Norway*.

*England.*—Webster, Stevens, Francis, Galt, [? America].

*Scotland.*—Coxe, Mitchell, Sibbald.

*France.*—Labitte, Jules Duval.

*Spain.*—Pjados.

*Poland.*—Plaskowski.

*Siebenburgen.*—Kellermann.

*Hanover.*—Droste.

With the exception of Schröder van der Kolk, who remained two days in Gheel, and Dr. Droste from Osnabruck, who stayed there some days, at different periods, and who is untiring in his pleadings for the place,* none of these gentlemen devoted more than a few hours to its examination. Be it further observed that, until now, some countries of Europe, such as Switzerland, Italy, &c., have never sent any visitors to Gheel; and others, as France, England, Austria, and the whole of Germany, have only despatched a few. Previous to its reorganization, Gheel was still more scantily inspected, for, from the most minute inquiries, we have only been able to trace the following:—Simorurt and a few others from Belgium. Hume and Morrison; Sir Andrew Halliday, 1828; Dr. Cumming, 1852; Dr. Browne, 1838; Morell, 1844, from England. From France—Esquirol and Voisin, Moreau de Tours, 1842; Brierre de Boismont, 1846; Ferrus, 1849. We were also informed by Drs. Grünty, of Leipsic, and Lessing, from Sonnenstein, in Saxony, that they had visited Gheel years ago. Under Parigot, from 1849 to the beginning of 1856, there were scarcely any visitors at all at Gheel, except Dr. Droste and Dr. Biffi from Milan, and the philanthropists, Appert, from Hamburg, Podista, from Italy, and lastly, Dr. Begley, from England. We have thus, with careful research, scarcely been able to enumerate fifty persons who have visited this institution for a few hours, a colony that has been for centuries in existence, and which, in recent times, has, both theoretically and practically, progressed with modern science. But this institution, which had been so rarely visited and so superficially examined and judged, not only maintains itself, but flourishes, improves, and increases. In the words of Dr. Damerow,† Gheel must be looked upon as an historical and practical basis for every reform in the science and administration of lunacy, and as having produced wonderful results. It therefore stands to reason that the discussion, both verbally and in writing, of this most important institution, must not be closed, but that it must be renewed, energetically followed up, and thoroughly criticised by competent men. All that has hitherto been written on Gheel is referred to in the notes added to our article.‡

---

* Refer to the *Correspondenz Blatt für Psychiatrie*, August 31, 1856; further, *Allgemeine Zeitschrift für Psychiatrie*, 1853, vol. x. part ii.; further, *Medicinische Aehrenlese* (Rakhorstische Burhh, Osnabruck), October, 1856, and January, 1859 and 1860; lastly, September, November, and December, 1860, of the same Aehrenlese.

† *Allgemeine Zeitschrift für Psychiatrie*, 1855, p. 443; 1856, p. 147; 1857, p. 491; 1858, p. 412.

‡ Whatever has been written on Gheel has been of a cursory kind; the colony has never been systematically examined nor criticised. Most has been done by Professor J. Parigot, of Brussels. He published several articles on Gheel in the

3. The third question was entirely ignored by the meeting at Eisenach, which was in conformity with their affirmation of the second question. As we have disputed that opinion, we are obliged to consider this last question also. We need wait no longer for further proofs of the favourable results obtained by the care of lunatics at Gheel, as we already possess, from the observations of a number of years, sufficient evidence of it, and we can only regret that Dr. Flemming seems unaware of this. The official reports of the present chief physician of Gheel, Dr. Bulcken—and a more industrious, well-qualified, and highly-informed scientific physician it would be difficult to find—which he periodically furnishes to the Minister of Justice and the permanent Commission appointed for the superintendence of the Gheel institution, supply us with the following results. From the beginning of the year 1856 to the end of 1859, 527 lunatics were received at Gheel, of whom 96 were discharged recovered; thus an average of fifteen per cent. cures is obtained. These results would appear still more favourable if the trouble were taken to investigate the special cases of recovery. It must not be forgotten that it is a principle with the Belgian Government, never to send a case to Gheel which has not been pronounced incurable, and that the communal physicians must be particularly careful only to recommend such cases to be transferred there.

It is not our intention to discuss here the diagnosis arrived at, but it is known from the official registers of the patients, that during the last four years only 145 have been pronounced curable of the 527 accepted; so we may conclude that from 527 patients 145 being deducted as curable and 96 of these being cured, the per-centage of recoveries reached 66·0. The affections of the cured patients were—

---

*Journal de Médecine de Brussels*, in the years 1850 to 1860; further, the article mentioned in a previous note, and several pamphlets. In 1852 he wrote his book, *L'air libre et la vie de la Famille dans la Commune de Gheel.* Bruxelles, Ternier, 1852.

The *Official Reports on Lunatic Asylums*, by the Inspecteur-General of lunatics at Brussels, Ducpetiaux, and the few words of Esquirol and Guislain, contain, in addition to the references already given, nearly everything that has been published on Gheel and its system. The Official Reports of the chief physician, Dr. Bulcken, which are almost unknown, furnish in reality the most complete material. We recommend these reports for consultation and examination. They are published by Hayer, at Brussels. Jules Duval has published an appendix to his work already mentioned, under the title *Gheel une Colonie*, in which we find a complete bibliography on this subject. Observations on Gheel occur in several journals and books on Psychiatry, particularly in Griesinger's *Lehrbuch du Psychiatrie*, p. 396, and in the *Lancet*, July 18, 1857, August, 1860, 4th, 11th, and 28th. Moreau de Tours has published his views on Gheel in the *Annales Medico-Psychologiques*, 1842, so likewise Brierre de Boismont in the same Journal, 1852, and 1846, in a separate pamphlet. Both these gentlemen are again going to visit Gheel, and we are very anxious for their renewed opinions on it.

| | | | |
|---|---|---|---|
| Mania . . . . . | 54 males, | 45 females. | Total, 99 |
| Hypochondriacal melancholy | 16 „ | 19 „ | „ 35 |
| Progressing imbecility . | 7 „ | 2 „ | „ 9 |
| Totals . . . | 77 | 68 | 145* |

The ages of the cured averaged from twenty to fifty years.

The district of Brussels, which furnishes the greatest portion of the lunatic population of Gheel, affords a still more powerful proof of the value of the colony.

Of 135 lunatics received during the last four years, two-thirds were pronounced radically incurable, yet thirty-five of them have already been sent away cured, which makes a per-centage of thirty-two cures per hundred. We repeat, we take these diagnoses as they come, and leave the separate cases to the respective responsibilities of the parties who formed them. Nor shall we repeat the constantly recurring inquiry how far we are to accept such statistical evidence, as we must of necessity take such facts for a basis of examination. We would, however, refer to the complete statistics furnished by the chief physician, Dr. Bulcken, in his reports, which are conscientiously compiled. We find the most minute changes of population, details of increase and decrease, cures, deaths, and other accidents reported to satisfy the present requirements of our science.

It would lead us too far to repeat and examine these specialities; moreover, they may be found in the appendix of Jules Duval's book on Gheel. We think we have already proved all we wished to do—viz., as it is a chief condition that only those are sent to Gheel who have been pronounced *incurable*, and the good effects of the care at Gheel are proved by many cures, we must consequently admit that the system of Gheel furnishes the most favourable results for the cure of lunatics. Professor Parigot, the most indefatigable advocate and constantly-assailed champion of Gheel,† has, therefore, only lately maintained what has long been proved. We may now ask, for what information is Dr. Flemming waiting, when he has already been furnished with abundant facts and innumerable proofs?

4. The reply to the first part (*a*) of this question has been so frequently given, that we shall not enter into its discussion; but one observation we cannot forbear making, namely, that in the construction of new asylums it is often the chief physician who originates more expenses, by recommending costly adornments of

* [There is an error in Dr. Munday's figures, which we have not the means to correct.—Ed.]

† Professor T. Parigot, of Brussels, has sacrificed his position, his time, fortune, and medical practice for the defence of Gheel and its system, and yet his professional brethren in phrenopathy hurl reproaches at him. Does it require a surer proof, that he defends a great cause with talent and truth?

the building. As to the second part of this question (*b*), referring to the formation of colonies as adjuncts to lunatic asylums, we are both surprised and delighted that this proposition has been thought of sufficient importance for a further discussion at the next meeting. We are astonished, for this simple reason, that we cannot understand that those who can logically reject the second question, and completely ignore the third, can yet reserve for discussion the very principle which is involved in the two rejected questions. The natural consequence must be that the next meeting will take up these two questions, examine and sift them in all their bearings, and prove to the world the thorough inconsistency of the Eisenach resolution.

On the subject of Dr. Flemming's fourth question, we shall give only a few words of a general nature.

First; the creation of lunatic colonies, patronal or family asylums, on which the lunatic asylums should depend, is, (*a*) practical in execution; (*b*) urgently necessary; (*c*) financially and administratively necessary; (*d*) therapeutically effective; and (*e*) they suffice for every desire for the protection and furtherance of order in society and sanitary police, and are satisfactory to the demands of humanity as well as those of modern science.

*a*. The best proof of the practical execution of the plan is found in the institution of Gheel, which has been established for centuries; and in spite of the various vicissitudes it has suffered, it has in recent times progressed more and more in science, so as to keep up to the demands of the age, and must be looked upon as the basis for the pressing reorganization of the healing art for lunatics.

*b*. As it cannot be denied that the entire sequestration of all lunatics which is still persevered in, is not only contrary to all the therapeutical laws of science, but also entirely opposed to every personal and social right, it stands to reason that the urgently recommended re-organization, which has been proved to be quite possible, is urgently necessary, and this urgency is strengthened by the reply to the first question.

*c*. It is well known what enormous sums the present palace-like buildings cost States in their construction and administration, and how, after a few years, they prove utterly inadequate to the necessities of the time, and require new sacrifices. We suppose therefore that, First, a large estate is bought, containing every requisite which a topographic, telluric, and social regard require for a good lunatic institution—in other words, the right situation, climate, air, light, water, land, and people for such an undertaking. Secondly; that in the centre of the estate a central asylum is built, which shall be in every respect complete and separated in two divisions. The first division for new and acute cases, the second for chronic cases. The latter would be sequestrated according to necessity, not only to prevent danger, and for personal protection, but also for therapeutic purposes and diagnostic examinations. Thirdly; that various cottages should be built on the estate, if there are not enough already present, which should contain the necessary requirements. Fourthly; in these cottages or homes those patients, either in the acute or chronic phases, who do not need sequestration, should be tended and protected; they should be properly fed, nursed, and treated by the inmates of these homes, if they are fit for the office, or if not, properly qualified persons and their families should be placed in them. Fifthly; to every one of these cottages and their inmates land, pasture, cattle, &c., must be given, of which they must take care, and for which they must pay rent to the estate. The steward, on the other hand, would receive pecuniary compensation for his patients, according to the plan or mode of division laid down. Sixthly; no house should contain, at the utmost, more than four patients. The separation of sexes is often necessary, but not indiscriminately so. Gheel furnishes proofs of this. Seventhly; the State must be purchaser and proprietor of the estate, but the temporal lord of the manor and principal manager of the estate would be the chief physician. Every officer of the administration and of the farm should be his subordinate, and every steward and farmer dependent on him alone. Eighthly; the number of the assistant physicians would depend on the size of the estate and lunatic population. At all events, the number should be much increased, and they should remain much longer in the asylums than they do at present. Ninthly; there should be a perfect code of laws for the guidance of the administration, the assistant-physicians, nurses, stewards, &c. Tenthly; various modifications of the laws will be necessary according to the land, population, and other exceptional circumstances. We now put the question, Would not the purchase of such an estate be financially and administratively advantageous to the State, considering the income it would derive from the patients, the farms, &c.? Our proposition would further have this advantage over the present system, that such an estate would, if properly managed, be a perpetual patronal lunatic asylum for many States, serviceable for ever, whilst now four or five costly establishments barely satisfy the demands on them. Larger countries would of course be obliged to establish several such estates in convenient positions. The first purchase capital would of course be greater than is at present necessary for the formation of a lunatic asylum, but it must not be forgotten that the capital for the latter is lost, while the sums for the estates not only pay a good interest, but are eventually paid up. We think that we have now proved, in outline, that this project is financially cheap, and administratively practical. Do not ask where such an estate is to be found, and where the tenants, directors, administrators, &c.? Those who thus ask prove at once their inefficiency and incapacity. It would be another matter to ask where are the promoters and protectors of such a scheme. These certainly would only be found in wise and enlightened kings and their advisers, and therefore necessarily all individual speculations and corrupt private asylums would receive their death-blow.

*d.* Therapeutical practice here distinguishes two principal and fundamental rules :—

1. Sana cito et jucunde.

2. Procura incurabilibus summum boni et animi quod licet et prosit.

This project includes both objects. Shall we draw a comparison between the situation and arrangements of the best-conducted private asylums, and those of the unjustly accused and calumniated Gheel? And yet we do not look upon Gheel either in a topographic or administrative sense as a model for our new institution; it should simply be an instructive example, whose good we would imitate, and whose defects we would avoid.* That patients are easily and quickly cured at Gheel, we have sufficiently proved in the third question. What results, then, might we not expect in cures, were all unfavourable influences removed, and we were furnished with means still further to effect cures?

*e.* The proofs furnished under *a, b, c, d* render it unnecessary to enter more minutely into this last point, as it is already settled by these arguments. Order, peace, and sanitary police are nowhere in greater efficiency than at Gheel. In a whole century there has not been one act of violence, and this in a place where there are 600 lunatics, which number is often increased to a population of 1000, who move freely in families, surrounded by women and children, who mix with strangers and inhabitants, work, amuse, and occupy themselves in the fields, woods, and meadows, and on the river. No suicides have happened here for years; no injuries, no wanton destruction of property, no incendiaries, and in the last ten years there have been scarcely any cases of rape. Here lunatics nurse children and are nursed by them, escapes or ill-usage from lunatics are matters of the greatest rarity. Shall we still ask whether humanity suffers under such management, or whether it derives benefit from it? Or shall such principles and their fruits remain solitary in the world, with no attempt to repeat

* We do not suffer from "Gheelnomania," as some one took upon himself, in a remarkable manner, to observe, and who subsequently acknowledged all at once the truth of our observations. Gheel is no model for us, but an instructive example.

or improve them? The theory of science absolutely demands practical reforms; the greatest authorities of our time, adversaries as well as advocates, unite in the demand for reforms. It is time, therefore, that we should throw off the stagnation under which we have laboured since the reforms of Pinel; it is time that we should no longer suffer humanity and the true laws of nature and science to be trodden under foot; we must no longer permit the caricatures which routine and custom have foisted upon us—disgraceful pictures which disgust us by their egotism, their fear, and their eagerness for gain. These are our views of the question stated in a few words. We have been requested by several parties to write them down, and we have endeavoured to do so freely and openly. After the publication of our systematic work, we shall wait for the scientific criticism of its practical value to improve and elucidate our views as may be necessary; we shall never, however, cease to expose incompetent criticism. We have one final wish, which is, that the scheme we have so untiringly advocated theoretically shall be practically carried out, for words without deeds are of no avail.

### POSTSCRIPT.

This article was written for the April number of the Psychological Journal, but forwarded at too late a period for insertion. Since its preparation a good deal of agitation has occurred respecting Gheel. Thus—

1st. Dr. Browne wrote an article adverse to Gheel in this Journal (April, 1861) p. 213 to 237.

2nd. Dr. Sibbald published an article in favour of Gheel in Dr. Bucknill's *Journal of Mental Science*, April part, 1861, p. 31 to 61.

3rd. Dr. Bulcken, chief physician at Gheel, has published his second official report on this patronal asylum.

4th. Dr. Parigot has protested in an article of the *Medical Journal of Brussels*, against the words used by the Medico-Psychological Society of Paris against Gheel.

5th. Dr. Bulcken also protested against these observations in a letter published in the April part of the *Annales Medico-Psychologiques*.

6th. Dr. Ferrus, of Paris, a celebrated and meritorious physician, who was the instigator of these protests, has since died.

7th. Moreau de Tours, Paris, wrote a critical article on Dr. Morel's new book, the *Non-Restraint*, in *L'Union Medicale*, February part, No. 25 and 26, and adds some very characteristic observations on Gheel.

8th. At a meeting held at London, on 19th April, 1861, for the erection of a "Benevolent Asylum for the Insane of the Middle Classes," Mr. Stephen Cave, M.P., spoke in favour of Gheel.

9th. The Gheel question was also mentioned by physicians, in a preliminary meeting of the society which will assemble at Speyer this summer.

10th. The director of the Lunatic Colony, Fitz James, in France, near Clermont (Oise), Dr. Gustave Labitte, has published a most remarkable pamphlet on this colony.

11th. The commissioners for the purpose of erecting new lunatic asylums for the Department of the Seine, which is Paris, have in effect decided for decentralization and for colonization. Dr. Girard de Cailleun is the medical referee of this commission.

---

